# Causes of Death in HIV Patients and the Evolution of an AIDS Hospice: 1988–2008

**DOI:** 10.1155/2012/390406

**Published:** 2012-05-17

**Authors:** Ann Stewart, Soo Chan Carusone, Kent To, Nicole Schaefer-McDaniel, Mark Halman, Richard Grimes

**Affiliations:** ^1^Casey House Hospice, Toronto, ON, Canada M4Y 2K8; ^2^Department of Family & Community Medicine, Faculty of Medicine, University of Toronto, Toronto, ON, Canada M5S 1A8; ^3^Department of Clinical Epidemiology and Biostatistics, McMaster University, Hamilton, ON, Canada L8S 4K1; ^4^Black Creek Community Health Centre, Toronto, ON, Canada M3N 3A1; ^5^Department of Psychiatry, St Michael's Hospital, Toronto, ON, Canada M5B 1W8; ^6^Centre for Research on Inner City Health, The Keenan Research Centre in the Li Ka Shing Knowledge Institute of St. Michael's Hospital, Toronto, ON, Canada M5B 1W8; ^7^Department of Psychiatry, Faculty of Medicine, University of Toronto, Toronto, ON, Canada M5S 1A8; ^8^Division of General Internal Medicine, The University of Texas Health Science Center at Houston, Houston, TX 77030, USA

## Abstract

This paper reports on the transformation that has occurred in the care of people living with HIV/AIDS in a Toronto Hospice. Casey House opened in the pre-HAART era to care exclusively for people with HIV/AIDS, an incurable disease. At the time, all patients were admitted for palliative care and all deaths were due to AIDS-defining conditions. AIDS-defining malignancies accounted for 22 percent of deaths, mainly, Kaposi sarcoma and lymphoma. In the post-HAART era, AIDS-defining malignancies dropped dramatically and non-AIDS-defining malignancies became a significant cause of death, including liver cancer, lung cancer and gastric cancers. In the post-HAART era, people living with HIV/AIDS served at Casey House have changed considerably, with increasing numbers of patients facing homelessness and mental health issues, including substance use. Casey House offers a picture of the evolving epidemic and provides insight into changes and improvements made in the care of these patients.

## 1. Introduction

Remarkable changes have taken place since AIDS was first described in 1981. The face and complexity of both living and dying with HIV have evolved significantly over the last three decades. Today, incidence is increasing in new subpopulations, new treatments are becoming available and individuals are living longer on treatment. There are also new and unanticipated complications of this virus which result in changing causes of death. In order to effectively research, plan, and provide the best care possible for people living with HIV/AIDS, it is essential that we understand both trends and the current state of the epidemic today, including differing causes of mortality.

In a classic paper examining deaths of individuals who were diagnosed with AIDS prior to 1986, the one-year mortality rate was 51.2% and the 5-year rate was 84.8%. The principal causes of death were *P. carinii* pneumonia (now referred to as *P. jirovecii *pneumonia) and Kaposi sarcoma in 82% of the cases [1]. By the mid-1990s antiretroviral combination therapy became available. These drugs suppressed HIV replication and, as a result, deaths due to HIV infection were greatly reduced. The number of deaths in HIV-infected persons in the United States dropped from approximately 50,000 in 1995 to 18,000 in 2008 [2]. AIDS deaths captured by the Public Health Agency of Canada, which counts only voluntary reporting of death in previously reported HIV/AIDS cases, significantly underestimates the number of AIDS deaths in Canada but shows a similar decline with 1501 deaths in 1995 and 53 in 2008 [3]. Effective medications also led to the near disappearance of many familiar manifestations of HIV. In 1994, over 95% of the deaths in HIV-infected persons in San Francisco were HIV related [4]. Tracking changes in death as a result of antiretroviral therapies, the San Francisco study [4] found, between 1994 and 1998, deaths from wasting in HIV infected persons declined from 252 to 44; deaths from cytomegalovirus infection from 274 to 34; from Kaposi sarcoma from 246 to 32; and from *P. carinii* from 187 to 29 [4]. Declines in these conditions, and other HIV-related conditions, have continued. A recent study of 1597 deaths that occurred in 13 cohorts between 1996 and 2006 found that over half of deaths in people with HIV/AIDS were not attributed to diagnoses that have been traditionally related to HIV infection. Of these, 23% were non-AIDS-defining malignancies; 16% were non-AIDS-defining infections; 15% were related to drugs and/or violence; 14% to liver disease [5].

The changes in prognosis and associated diagnoses have resulted in a new burden of care on organizations that serve HIV-infected persons. One type of healthcare organization dramatically affected is the hospice that initially served those dying with HIV. In the early years of the epidemic, individuals arrived at these institutions with a devastated immune system that resulted in multiple HIV-related opportunistic infections and death within months. With the advent of combination antiretroviral therapy and an increased understanding of the clinical and medical management of HIV-related conditions, HIV/AIDS patients are now frequently discharged to return home to their community. However, this population requires a range of healthcare services to manage the extensive medication regimes and complex medical and psychiatric comorbidities, many of which are non-AIDS defining [6–9]. With the decline in the number of deaths in HIV-infected persons, many AIDS hospices have closed, evolved, or expanded to provide rehabilitation care. Thus, the changes in hospice care over the last 30 years provide a bellwether, giving an insight into the transformation that has occurred in HIV care.

As a means of tracing this historical change, we performed a retrospective study to compare the causes of death of those who died in a Toronto hospice, known as Casey House, between 1988 and 2006–2008. These two time-periods were chosen to represent the first year of operation of the hospice, before the development of antiretroviral therapies, and a recent portrayal of patients in a time where highly active antiretroviral therapies (HAART) are widely available in developed countries. A longer time-frame in the post-HAART period was necessary to capture similar numbers of deaths. In another report we have described the demographics and medical, and psychiatric comorbidity of the entire population of patients admitted to the facility in 2008 [10]. We include some of that data here to provide broader context of the morbidity as well as mortality currently experienced by the patients needing care.

## 2. Methods

### 2.1. Setting

Casey House was originally established in 1988 as Canada's first hospice for individuals with HIV infection. Today, Casey House is a 13-bed hospital which provides both in-patient and home care services to people living with HIV/AIDS. There are approximately 100 in-patient admissions a year. Individuals with HIV may be admitted for sub-acute rehabilitative care, medical and psychiatric symptom control (including pain management), post-hospitalization support, end-of-life palliative care, or respite care. Care is provided by an inter-professional team including primary and consultant specialist physicians, nurses, social workers, and rehabilitation therapists.

### 2.2. Data Collection and Analysis

A retrospective review of patient charts and death certificates was undertaken at Casey House, Toronto, Canada. The first set of the analyses focused on patients who had died at Casey House. We defined the first 10 months of Casey House operation as the “pre-HAART” period, thus including records of all patients who had died between the first day Casey House opened its doors, March 1, 1988, and December 31, 1988. In this era, Casey House operated solely as a hospice and patients were admitted only for end-of-life care. We compared characteristics of these pre-HAART patients who died to all Casey House patients who died during a 36-month time period from January 1, 2006 to December 31, 2008. The years 2006–2008 were chosen to represent the current epidemic in an environment where highly-active antiretroviral therapies are widely available. We describe differences in causes of death in the two time-periods. Uneven time-periods were necessary to obtain comparable numbers of deaths in each time.

In addition to focusing on patients who died during their tenure at Casey House, we describe the characteristics of all patients admitted to Casey House during the entire year of 2008 (January 1–December 31) to demonstrate how the facility had evolved as the HIV epidemic, and consequently survival, changed [10].

Clinical and sociodemographic characteristics were collected from patient charts and death certificates. Due to developments in medical knowledge and changes in the scope of practice, not all variables were available or collected for all deaths. Causes of death and types of cancer death were identified and classified using the International Classification of Diseases, the 10th edition. Causes of death were identified as AIDS-defining conditions according to the Centers for Disease Control and Prevention (CDC) [11]. Malignancies were identified as AIDS-defining malignancies (including all malignancies listed by the CDC as an AIDS-defining condition: Kaposi sarcoma, Burkitt lymphoma (or equivalent term), immunoblastic lymphoma, lymphoma (primary of brain), and invasive cervical cancer) or non-AIDS-defining malignancies. Simple descriptive and chi-square statistics were used to analyze the data.

This research project was approved by the Research Ethics Board at St. Michael's Hospital, Toronto, Canada.

## 3. Results

### 3.1. Patient Characteristics

**Pre-HAART Era**

Fifty-nine patients died at Casey House in the 10 months between March 1 and December 31, 1988. All patients were male and all but one individual (98.3%) self-identified as men having sex with men (MSM). Individuals lived an average of 1.5 years (SD = 0.9) with an HIV/AIDS diagnosis before dying. The mean age at death was 39.0 years (SD = 9.3).

**Post-HAART Era**

Forty-eight patients died at Casey House in the 36 months between January 1, 2006 and December 31, 2008. Forty-three (89.6%) patients who died were male. Thirty-four (80.9%) persons identified MSM. The average age at death was 48.1 years (SD = 8.6) after living an average of 13.5 years (SD = 6.5) since HIV/AIDS diagnosis.

Characteristics of patients who died in the pre- and post-HAART period are summarized in Table 1. In the pre-HAART era 37.2% of patients had mental health issues, this almost doubled in the post-HAART era to 67.4%. In 1988, only 5.3% were homeless, however the number of homeless and socioeconomically marginalized patients increased significantly in the post-HAART era. In terms of substance use, the number of smokers remained relatively constant at approximately 50%. In the pre-HAART era, 21.4% endorsed recreational drug use and 7.3% reported injecting drugs. The self-reported incidence of both behaviours nearly tripled in the post-HAART era to 63.8% and 23.4%, respectively.

### 3.2. Cause of Death

**Pre-HAART Era**

The primary cause of death was identified as an AIDS-defining condition in all 59 deaths in 1988. Twenty-two percent (*n* = 13) were due to AIDS-defining malignancies. Forty-two percent (*n* = 25) of deaths were due to AIDS-defining opportunistic infections, most commonly disseminated *Mycobacterium avium* complex disease (MAC) (*n* = 5), toxoplasmosis (*n* = 5), and *Pneumocystis* pneumonia (PCP) (*n* = 3). Thirty-six percent (*n* = 21) of deaths were identified as other AIDS-defining conditions. In 19 of these cases the cause of death was listed only as “AIDS.”

**Post-HAART Era**

Forty-eight percent of deaths in the post-HAART era were attributed to AIDS-defining conditions. Thirty-one percent (*n* = 15) of individuals died of AIDS-defining opportunistic infections; 6% (*n* = 3) from AIDS-defining malignancies; 10% (*n* = 5) from other AIDS-defining conditions. The remaining 25 deaths (52%) were attributed to: non-AIDS-defining malignancies (*n* = 13; 27%); liver disease (*n* = 3; 6%); other causes (*n* = 9; 19%) including multi-organ failure (*n* = 3) and respiratory failure (*n* = 2). Fourteen patients had hepatitis B or C listed as a contributing cause of death.

**Figure 1:**
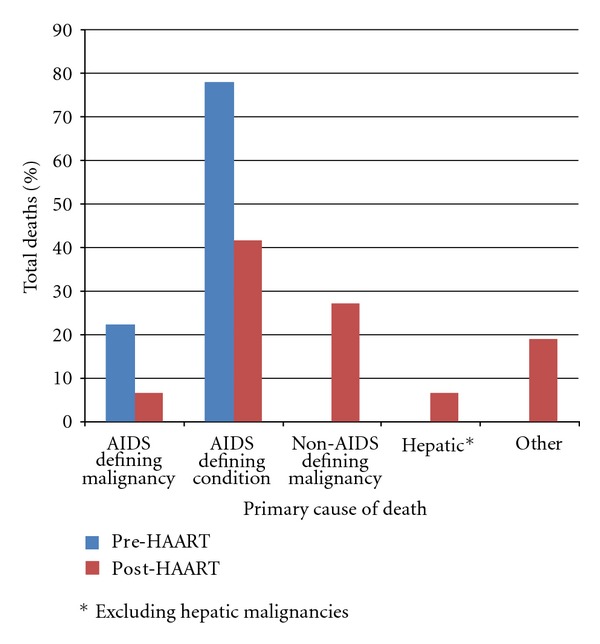
Primary cause of death in individuals who died at Casey House in 1988 (pre-HAART) in comparison to 2006–2008 (post-HAART).

Figure 1 presents a summary and comparison of the primary causes of death in the two time periods.

### 3.3. Malignancies

**Figure 2:**
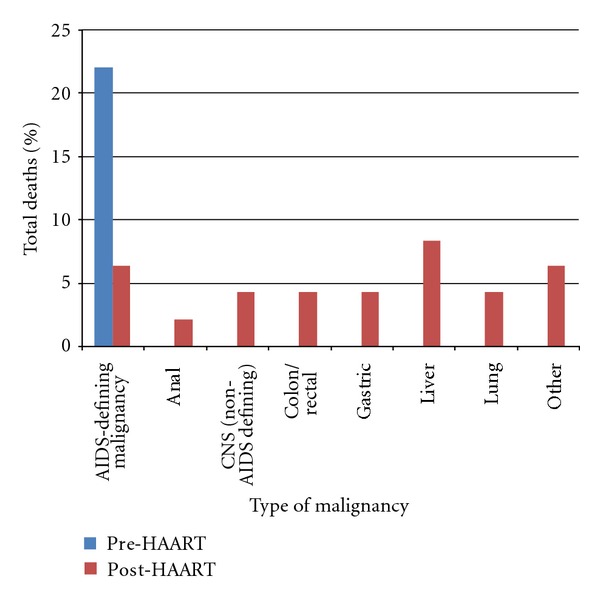
Types of malignancy in individuals who died at Casey House in 1988 (pre-HAART) compared to 2006–2008 (post-HAART).

Malignancies accounted for 22% (*n* = 13) of deaths in the pre-HAART era. All of these were AIDS-defining malignancies: 11 were attributed to Kaposi sarcoma and 2 to lymphoma. An additional 4 deaths had Kaposi sarcoma listed as a contributing factor. In the post-HAART era, malignancies accounted for the primary cause of death in 33% (*n* = 16) of deaths: 6% (*n* = 3) were due to AIDS-defining malignancies and 27% (*n* = 13) were due to non-AIDS-defining malignancies. Kaposi sarcoma was not listed as the primary cause of death for any patients in the post-HAART period but was a contributing factor in one death.  Figure 2 illustrates the frequency of different types of malignancies present in individuals at the time of death for the two time-points. This figure includes three individuals in the post-HAART period that had a malignancy listed as a contributing cause of death but not as the primary cause of death.

In the post-HAART era, many of the non-AIDS-defining malignancies occurred in patients who were on HAART therapy, with reasonably well-controlled disease. Admission CD4 count was available on ten patients who died from non-AIDS-defining malignancies, of whom 70% had a CD4 count greater than 200 cells/mm^3^. CD4 counts ranged from 25–800 cells/mm^3^. Of the eight patients who had admitting viral loads, six were undetectable, and the other two had 150 and 400,000 copies/mL.

### 3.4. HIV/AIDS Patients Admitted in 2008

Between January 1 and December 31, 2008, 87 individuals were admitted to Casey House. Of the 87 individuals who were admitted, 17 (*n* = 19.5%) died during their stay at Casey House: seventy-percent of whom had a CD4 count below 200 cells/mm^3^. Of the individuals who died with decreased immune system function, 25% were homeless and 25% lived in supportive housing. All individuals who died with a CD4 count above 200 cells/mm^3^ reported renting their own housing (excluding supportive housing).  Table 2 includes descriptive data for all patients admitted in 2008 and compares individuals who died during their stay to those who were discharged. There were no statistically significant differences (at the *P* < .05 level) between patients who died and those who were discharged, although there were slightly more individuals with a CD4 count below the median in those who died (*P* = .07). Eighty percent of patients admitted were male. The average age at admission was 48.9 years (SD = 10.5) and the median CD4 count was 150 cells/mm^3^. The average number of medical comorbidities experienced by patients at the time of admission was 5.9 (SD = 2.3). The most common being: AIDS-defining opportunistic infections (*n* = 27; 65.9%); respiratory conditions, such as chronic obstructive pulmonary disease, (*n* = 16; 39.0%), and non-AIDS-defining malignancies (*n* = 10; 24.4%).

## 4. Discussion

Looking at the evolution in rate and cause of death in a hospice, over a twenty-year period, gives important insight into the development of the HIV epidemic. In 1988, HIV infected persons admitted to this facility were suffering from a ravaged immune system that resulted in deaths caused by a spectrum of diagnoses defined as AIDS-related conditions. All admissions were for palliative care. At the time, AIDS was felt to be a hopeless diagnosis. Casey House embraced the caring and compassionate hospice care model—often demedicalizing the treatment of the patients, and as a result specific investigations were not made into the particular cause of death and many certificates simply state this patient died of “AIDS.” In contrast, by 2008 less than 20% of patients admitted to Casey House died during their stay. And thus, 80% were discharged, often following an in-patient stay which included investigations and treatment for HIV as well as management of medical comorbidities and mental health issues. This highlights a significant change in the type of care needed by patients and also the lived experience of HIV-positive individuals.

Over the 20-year period examined in this study, there were significant changes in both the primary cause of death and the characteristics of those who died. In 1988, all deaths were attributed to an AIDS-defining condition, while less than half were in the 2006–2008 time period. Malignancies were a common cause of death in both time periods, although the types of malignancy changed considerably. Although the population remained largely male and mostly among men who have sex with men, in the post-HAART period 10% of deaths were in women. Individuals who died in the post-HAART period also reported greater IV drug and recreational drug use and more individuals were identified as homeless and with a mental illness.

Surveillance data and other research reports comparing the pre-HAART and post-HAART era show similar changes in the demographics of the HIV epidemic and causes of death. Although HAART has significantly decreased mortality in HIV-positive individuals, mortality is still significantly higher than in the general population and there are subgroups that are at substantially greater risk such as injection drug users [5]. Studies linking HIV/AIDS databases and cancer registries over approximately the same time period have shown a dramatic decrease in AIDS-defining malignancies and an increase in non-AIDS-defining malignancies [12, 13]. As experienced at Casey House, the most common cancers of the pre-HAART era were Kaposi sarcoma and non-Hodgkin lymphoma. Both are typically linked with low CD4 count and coinfection with viral agents. Although such AIDS-defining malignancies remain prevalent in the HIV/AIDS population in the post-HAART era, numbers of non-AIDS-defining malignancies have increased as much as 20% in the US [14]. Between 2001 and 2005, the most common types of non-AIDS-defining cancers were lung cancer, anal cancer, liver cancer, and Hodgkin lymphoma [13]. This is reflected at Casey House. The Swiss cohort study notes that certain non-AIDS-defining malignancies associated with smoking such as lip, mouth, pharynx, and lung cancers increased in the post-HAART era. In addition, cancers of the liver associated with Hepatitis B and/or Hepatitis C coinfection, as well as human papilloma virus-related cancers, such as anal cancer, were also increased. It was speculated that living longer with disease, combined with partial immune reconstitution, allowed long-latency cancers to progress and manifest [12].

The studies make the point that people living with HIV/AIDS are at greater risk of cancer than the general population, and there is a great deal of speculation as to why this occurs. Increased cancer risk is believed to be a complex interaction, with multiple factors, including impaired immunity, co-infection with biologic agents carcinogenic to humans (such as HPV, EBV, and hepatitis), aging, HAART, and traditional risk factors for cancer, such as smoking and sun exposure [15]. There is some evidence that prolonged immunosuppression—as assessed by CD4 nadir—is independently linked to increased incidence of non-AIDS-defining malignancies [16]. In addition, the presence of HIV is linked to cytokine dysregulation and this may also play a role in increasing cancer risks [15].

Certainly there are complex factors at play in the patients seen at Casey House, who may have as many as 6 medical comorbidities, including coinfection with hepatitis, as well as risk factors such as unstable housing, injection drug-use, and smoking (approximately 50% of our patients smoke in comparison to 21% in individuals over 12 in Canada [17]). Individuals are now living for more than a decade with HIV and although some have maintained CD4 counts above 200, with suppressed virus, many individuals are not on HAART therapy or do not adhere to their medication regimes. It is interesting to note that patients with reconstituted immune systems remain at risk from cancer [18]. And, as reflected in the broader epidemic, our patients include more marginalized individuals who have significant social determinants of health risk factors such as homelessness and mental illness and this association also places them at risk [6, 7, 19]. In response, we need to adopt effective health promotion models for HIV-infected individuals and the health care response must be comprehensive and collaborative. Research and service initiatives should expand their focus to address medical preventive care including cancer screening and treatment initiatives such as smoking cessation, as well as social and psychosocial needs. These services and supports must be available in various settings to address the spectrum of needs, from case management and chronic symptom management to acute medical interventions and end-of-life care.

We acknowledge that this retrospective study has several important limitations. Data extracted from charts are susceptible to variations in reporting and diagnostic criteria and missing data are common. The changes in reporting practices are amplified in this study where data were collected from time-points 20 years apart in a disease that has been described for only 30 years. In 1988, many primary causes of death were written as “AIDS.” It is most likely that these individuals and deaths would have been evaluated and recorded differently today. This may underestimate the number of individuals who died of AIDS-defining malignancies and possibly other non-AIDS-related causes of death. In this study, death certificates and primary cause of death were identified for all individuals but data were incomplete for many clinical and demographic variables. Our ability to clinically describe these patients is also limited by the state-of-knowledge and treatments available in 1988. Many tests routinely issued to HIV-positive individuals today, such as CD4 counts and viral loads, either did not exist or were not widely used in 1988. Hepatitis C was not yet identified [20]. These findings are strengthened however by the complete inclusion of deaths that occurred at a single institution during the two periods of study. The description of all patients admitted in 2008 provides further context to the changes in the epidemic and the health care services provided. However, because Casey House is not an acute care hospital our study will not accurately represent the occurrence of certain types of death such as cardiac arrest, suicide, and trauma. It is also possible that individuals were differentially referred to Casey House in the two time periods as clinical knowledge and stigma of the disease evolved.

## 5. Conclusion

In summary, our findings are consistent with other studies in identifying changing trends in both the demographics and causes of death in individuals dying from HIV/AIDS. This study focuses on the patients and care provided at an AIDS hospice that evolved with the epidemic into a subacute hospital, giving some perspective to the ensuing changes that have occurred in organizations and the care supporting individuals with HIV/AIDS. As our knowledge and treatments for HIV improve, individuals are living longer but often with substantial psychosocial risk factors and significant medical and psychiatric comorbidities. Fewer HIV-positive individuals are dying from AIDS-defining conditions and increasing numbers of individuals are dying from non-AIDS-defining malignancies and other non-AIDS-defining conditions such as liver disease. The palliative care model of symptom management remains important for these patients. In order to plan and provide the care that is needed, it is crucial that we address both the medical and psychiatric complications and the social and psychosocial needs and risks of living with a chronic disease that has such a devastating and stigmatizing history. And, as long as HIV has no cure, we must also continue to acknowledge and provide care for individuals with HIV that embraces the palliative care model.

## Figures and Tables

**Table 1:** Demographics of patients who died in the pre-HAART and post-HAART periods.

	Pre-HAART (1988) (*n* = 59)	Post-HAART (2006–2008) (*n* = 48)	Statistical difference
Average age at death (*n* = 59; *n* = 48)	39.0 (SD = 9.3; range 23–65)	48.1 (SD = 8.6; range 30–69)	*t*(105) = 5.2, *P* < .001

Gender (*n* = 59; *n* = 48):			*x* ^2^(1) = 6.4,
Male	59 (100%)	43 (89.6%)	*P* = .001
Female	0	5 (10.4%)	

MSM (*n* = 59; *n* = 42)	58 (98.3%)	34 (80.9%)	*x* ^2^(1) = 9.1, *P* < .01

Average number of years since HIV/AIDS diagnosis until death (*n* = 59; *n* = 48)	1.5 (SD = .9; range 0–4)	13.5 (SD = 6.5; range 1–25)	*t*(47) = 12.6, *P* < .001

Average number of days in hospital prior to death (*n* = 59; *n* = 48)	31.6 (SD = 29.8; range 1–159)	44.8 (SD = 57.9; range 0–228)	*t*(67) = 1.4, *P* = .16

Has mental illness (*n* = 43; *n* = 46)	16 (37.2%)	31 (67.4%)	*x* ^2^(1) = 9.4, *P* = .002

Homeless (*n* = 38; *n* = 47)	2 (5.3%)	7 (14.9%)	*x* ^2^(1) = 2.1, *P* = .15

Current smoker (*n* = 56; *n* = 46)	28 (50.0%)	25 (54.3%)	*x* ^2^(1) = .19, *P* = .66

Recreational drug use (*n* = 56; *n* = 47)	12 (21.4%)	30 (63.8%)	*x* ^2^(1) = 19.0, *P* < .001

Current IV drug use (*n* = 55; *n* = 47)	4 (7.3%)	11 (23.4%)	*x* ^2^(1) = 5.7, *P* = .017

**Table 2:** Patient characteristics for all admissions in 2008.

	All patients(*n* = 87)	Patients who died (*n* = 17)	Patients who survived (*n* = 70)	Statistical difference
Average age (*n* = 17; *n* = 70)	48.9 (SD = 10.5)	49.4 (SD = 10.1)	48.8 (SD = 10.7)	*t*(85) = .22, *P* = .83

Gender (*n* = 17; *n* = 70)				
Male	70 (80.5%)	14 (82.3%)	56 (80.0%)	
Female	17 (19.5%)	3 (17.6%)	14 (20.0%)	*x* ^2^(1) = .05, *P* = .83

Median CD4 at admission (*n* = 16; *n* = 67)	150.0	68.8% below median	43.3% below median	*x* ^2^(1) = 3.4, *P* = .07

CD4 admission categories (*n* = 16; *n* = 67)				
<200	48 (57.8%)	12 (75.0%)	36 (53.7%)	*x* ^2^(2) = 2.9, *P* = .24
200–500	24 (28.9%)	2 (12.5%)	22 (32.8%)	
>500	11 (13.2%)	2 (12.5%)	9 (13.4%)	

Average number of comorbidities (*n* = 17; *n* = 70)	5.9 (SD = 2.3)	5.6 (SD = 2.2)	5.9 (SD = 2.3)	*t*(85) = .53, *P* = .60

AIDS-defining malignancy (*n* = 17; *n* = 70)	5 (5.7%)	2 (11.8%)	3 (4.3%)	*x* ^2^(1) = 1.4, *P* = .24

Non-AIDS defining malignancy (*n* = 17; *n* = 70)	14 (16.1%)	5 (29.4%)	9 (12.9%)	*x* ^2^(1) = 2.8, *P* = .10

Psychiatric disorder (*n* = 17; *n* = 70)	80 (92.0%)	14 (82.4%)	66 (94.3%)	*x* ^2^(1) = 2.6, *P* = .11
Cognitively impaired	41 (47.1%)	9 (52.9%)	32 (45.7%)	*x* ^2^(1) = .29, *P* = .59
Substance use disorder	36 (41.4%)	5 (29.4%)	31 (44.3%)	*x* ^2^(1) = 1.2, *P* = .26
